# Six Discourses, Delivered by Sir John Pringle, Bart. When President of the Royal Society; on Occasion of Six Annual Assignments of Sir Godfrey Copley's Medal. To Which Is Prefixed the Life of the Author

**Published:** 1784

**Authors:** 


V. *Six Diſcourſes, delivered by Sir John Pringle, Bart. when Preſident of the Royal Society; on occaſion of ſix annual aſſignments of Sir Godfrey Copley's Medal. To which is prefixed the life of the Author, by* Andrew Kippis, D. D. F. R. S. *and* S. A. 8vo. Cadell, *London*, 1783. 384 pages.

THESE celebrated diſcourſes *on the different kinds of Air—on the Torpedo—on the Attraction of mountains—on ſome late Improvements of the means for preſerving the health of mariners—on the Invention and Improvements of the reflecting teleſcope*—and *on the Theory of gunnery*, do not come within the plan of our Journal, if we except the firſt and the fourth; and with theſe, when we conſider the length of time that has elapſed ſince they firſt made their appearance, we may reaſonably ſuppoſe our readers to be well acquainted. It is on account of the elegant account it contains of the learned author, that we introduce this volume to our readers. We have long long wiſhed to communicate to them ſome authentic anecdotes of this deſervedly celebrated phyſician, and we cannot do this more ſatisfactorily than by availing ourſelves of the candid and accurate information here given us by Dr. Kippis.

Sir John Pringle, we are told, was born at Stichel-houſe in the county of Roxburgh, on the 10th of April 1707. He was the youngeſt of ſeveral ſons of Sir John Pringle of Stichel, Bart. His mother was ſiſter to Sir Gilbert Eliot of Stobs, Bart. Both the families from which he deſcended were very antient and honourable ones in Scotland. After purſuing his academical ſtudies at St. Andrew's and Edinburgh, he went to Amſterdam in 1728, being intended for the mercantile line; but his mind was turned to phyſic, by accidentally hearing at Leyden a lecture of Boerhaave's, which ſtruck him in a remarkable manner. From that time he applied himſelf diligently to medical ſtudies, and on the 20th of July 1730, was admitted to the degree of doctor of phyſic. His inaugural diſſertation was "*De marcore ſenili.*" During his reſidence at Leyden he contracted an intimate friendſhip with Van Swieten, who was not only his fellow-ſtudent, but alſo his phyſician, when he he happened to be ſeized there with a fit of ſickneſs. Nevertheleſs he did not owe his recovery to his friend's advice; for Van Swieten having refuſed to give him the bark, another preſcribed it, and Mr. Pringle was cured. From Leyden, Dr. Pringle went to complete his medical ſtudies at Paris, and ſoon after ſettled as a phyſician at Edinburgh, where, in March 1734, he was appointed profeſſor of pneumatics and moral philoſophy. In diſcharging the duties of this new employment, his text book was Puffendorff *De officio hominis et civis.* Beſides this he annually delivered ſeveral lectures on the immateriality and immortality of the ſoul; ſubjects which were not a little diſcuſſed at that period.

He remained at Edinburgh till 1742, when, by the intereſt of the earl of Stair, to whom he had been recommended by Dr. Stevenſon, an eminent phyſician at Edinburgh, he was conſtituted phyſician to the military hoſpital in Flanders. With this appointment he was permitted to retain his profeſſorſhip, and Meſſrs. Muirhead and Cleghorn were allowed to teach in his abſence.

At the battle of Dettingen Dr. Pringle was in a coach with Lord Carteret during the whole time of the engagement, and the ſituation they were were placed in was dangerous, as they were betwixt the fire of the line in front, a French battery on the left, and a wood full of huſſars on the right.

Soon after this his friend the earl of Stair retired from the army. Dr. Pringle offered to reſign with his noble patron, but that liberal minded commander would not permit him to think of it for a moment. He therefore continued to attend the army, in Flanders, through the campaign of 1744, and in the ſpring following, the duke of Cumberland appointed him phyſician general to his majeſty's forces in the Low Countries. He now thought it right to reſign his profeſſorſhip in the univerſity of Edinburgh, and in 1745 he was recalled from Flanders to attend the troops which were to be ſent againſt the rebels in Scotland. On the 30th of October of that year he was elected a fellow of the Royal Society.

In 1747 and 1748 he again attended the army abroad; but after the treaty at Aix la Chapelle came to reſide in London, and in April 1749 was appointed phyſician to his R. H. the duke of Cumberland. In 1750 he publiſhed his Obſervations on the Jail or Hoſpital Fever, which were afterwards incorporated in his great work on on the Diseases of the Army. In the same year he began to communicate to the Royal Society his experiments upon septic and antiseptic substances, which procured him the honour of Sir Godfrey Copley's gold medal. He afterwards communicated to that learned body several other papers, which appear in different volumes of their Transactions; and he likewise published in the Edin. Med. Essays, vol. V. an Account of the success of the *vitrum ceratum antimonii.*

On the 14th of April 1742 he married Charlotte, the second daughter of Dr. Oliver, an eminent physician at Bath. This connection, which we have heard was not a happy one (tho' this circumstance is not mentioned by the learned and worthy biographer) did not last long; the lady dying in the space of a few years.

In the same year Dr. Pringle published the first edition of his "Observations on the Diseases of the Army." Of this work, which hath now passed through seven editions, Dr. Kippis very properly observes, that it hath placed the writer of it in a rank with the famous Sydenham. "Like Sydenham, too—says he—he hath "become eminent, not by the quantity, but "the value of his productions; and hath afforded a happy instance of the great and de-

"served

" ſerved fame which may ſometimes ariſe from

" a ſingle performance."—

In the war that began in 1755, Dr. Pringle attended the camps in England during three ſeaſons. In 1758 he entirely quitted the ſervice of the army, and on the 5th of July in that year was admitted a licentiate of the college of phyſicians of London. In 1761 he was appointed phyſician to the queen's houſhold. In 1763 he was conſtituted phyſician extraordinary, and the year following (on the deceaſe of Dr. Wollaſton) phyſician in ordinary to her majeſty. In 1766 he was raiſed to the dignity of a baronet. In 1768 he was appointed phyſician in ordinary to the late princeſs of Wales, and in 1774 he received the laſt promotion that was given him in his medical capacity; which was his being made phyſician extraordinary to his majeſty.

In 1772, on the deceaſe of James Weſt, Eſq. Sir John Pringle was elected preſident of the Royal Society: this was undoubtedly the higheſt honour that he ever received. " It was at a very

" auſpicious time—Dr. Kippis obſerves—that Sir

" John Pringle was called upon to preſide over

" the Royal Society. A wonderful ardour for

" philoſophical ſcience, and for the advance-

" ment of natural knowledge, had of late years

" diſplayed itſelf through Europe, and had ap-

" peared with particular advantage in our own

" country.——The ſpirit of experimental inveſ-

" tigation was high; and nothing could be

" more agreeable to the genius of Sir John

" Pringle, than to cheriſh ſuch a ſpirit. He

" endeavoured to do it by all the methods that

" were in his power; and he happily ſtruck

" upon a new way to diſtinction and uſefulneſs,

" by the diſcourſes which were delivered by

" him on the annual aſſignment of Sir Godfrey

" Copley's medal." Dr. Kippis points out the particular merits of each of theſe excellent diſcourſes.

Several marks of literary diſtinction had been conferred on Sir John Pringle, before he was raiſed to the Preſident's chair. But after that event almoſt all the learned ſocieties in Europe were anxious to enroll him in the liſt of their members. Of the academical honours, enumerated by the learned biographer, we ſhall content ourſelves with mentioning that conferred by the Royal Academy of Sciences at Paris, who, in 1778, elected Sir John Pringle to ſucceed the celebrated Linnæus, as one of their foreign members. This honour, being limited to eight perſons, is juſtly eſteemed a moſt eminent mark of diſtinction.

As Sir John Pringle was in his ſixty-ſixth year when he was elected Preſident of the Royal Society, it was natural to expect that in the courſe of a few years the various and important duties of this ſtation would become burthenſome to him. A fall, from which he received conſiderable hurt, and which in its conſequences affected his health and ſpirits, likewiſe contributed, we are told, to induce him to reſign the chair. It hath indeed been reported that he was much hurt by the diſputes of the Society concerning electrical conductors; but Dr. Kippis, who was then in the habit of a ſtrict intimacy with Sir John Pringle, never heard from him any ſuggeſtion of this kind. His declining years, and the general ſtate of his health, form ſufficient reaſons for his reſignation, which took place in 1778, when Joſeph Banks, Eſq. (now Sir Joſeph Banks, Bart.) was unanimouſly elected Preſident in his room, "a gentleman," ſays our candid and learned biographer, "who had "eminently diſtinguiſhed himſelf by his ac-"quaintance with natural hiſtory; who had "failed round the globe, and performed "other voyages in purſuit of that branch of

" ſcience; who is preparing, at an immenſe

" expence and labour, the nobleſt and moſt

" ſplendid botanical work, which hath ever

" been preſented to the public; and who hath

" amply juſtified the choice that was made of

" him, by his attention to every part of his

" duty, and his aſſiduous concern to promote

" the intereſt and honour of the Society."

Though Sir John Pringle now did not attend the meetings of the Royal Society ſo conſtantly as he had formerly done, his houſe ſtill continued to be the reſort of ingenious men. He was held in particular eſteem by eminent and learned foreigners, none of whom came to London without viſiting him, and it would have been an uncommon thing not to have ſeen ſome of them at his Sunday evening converſations.

In the ſummer of 1780 he made an excurſion to Scotland, and purchaſed a houſe at Edinburgh, where he intended to paſs the remainder of his days. When he returned to London in the autumn, he ſet about preparing to put his ſcheme in execution. Accordingly he diſpoſed of his houſe in Pall-mall, and of the greateſt part of his library, and in the ſpring of 1781 removed to Edinburgh. " In this city," Dr. Kippis obſerves, " he was treated by perſons of

" all

" all ranks, with every mark of reſpect. But " Edinburgh was not now to him what it had " been in early life——many, if not moſt, of " his old friends and contemporaries were dead ; " and, tho' ſome of them remained, they could " not meet together with the ſame ſtrength of " conſtitution, the ſame ardour of purſuit, the " ſame animation of hope, which they had for- " merly poſſeſſed. The younger men of eminence " paid him the ſincereſt teſtimonies of eſteem " and regard ; but it was too late in life for " him to form new habits of cloſe and intimate " friendſhip." He likewiſe found the air of Edinburgh too cold for his frame. Theſe evils were increaſed by his increaſing infirmities, and he determined to return once more to London.

Before he quitted Edinburgh he preſented ten volumes, folio, of medical and phyſical obſervations, in MS. to the College of Phyſicians, on condition that they ſhould neither be printed, nor lent out of the library.

He came back to London in September 1781, and reſided in King-ſtreet, St. James's Square, where he renewed his Sunday evening converſations. The other evenings of the week he uſually paſſed with a philoſophical ſociety, of which which he had long been a member, and which met at Mr. Watſon's a grocer in the Strand. In the mean time, however, his ſtrength was declining with rapidity, and on Monday the 14th of January 1782, being with the ſociety at Watſon's, he was ſeized with a fit, which carried him off on the Friday following, in the 75th year of his age. On the 7th of February, he was interred in St. James's Church, Weſtminſter.

Dr. Kippis adds that, as a teſtimony of regard to his memory, at the firſt meeting of the College of Phyſicians at Edinburgh after his deceaſe, all the members appeared in deep mourning.

Sir John Pringle bequeathed the bulk of his fortune to his nephew and heir Sir James Pringle of Stichel, Bart: but a ſum, equal to ſeven hundred pounds a year, was appropriated to annuities, revertible to that gentleman at the death of the annuitants. He provided likewiſe for two ſervants, who had lived with him a conſiderable time; and he left legacies to Dr. Kippis and ſome other particular friends.

We come now to the character of Sir John Pringle.—Dr. Kippis conſiders him firſt as a practical phyſician. "He was diſtinguiſhed," we we are told, " in this respect, by his attention " and sagacity. For the recovery of his pa- " tients he was anxiously concerned ; and his " anxiety might, perhaps, be increased from " his conviction, that the art of physic, though " eminently useful, must ever, from unavoid- " able circumstances, be attended with a cer- " tain degree of uncertainty. His care was " rewarded with much success in the course of " his practice. In the exercise of his profes- " sion, he was not rapacious ; being ready, " on various occasions, to give his advice with- " out pecuniary views. This he never denied " to the poor ; and from many of his friends " in better circumstances, and who were well " able to afford the customary gratifications, " he refused to accept of fees.

" The turn of Sir John Pringle's mind," continues our author, " led him chiefly to the " love of science, which he built on the firm " basis of fact. With regard to philosophy in " general, he was as averse to theory, unsup- " ported by experiments, as he was with respect " to medicine in particular. Lord Bacon was " his favourite author ; and to the method of " investigation, recommended by that great " man, he steadily adhered. Such being his " intel-

" intellectual character, it will not be thought

" surprising, that he had a dislike to Plato.

" The speculations of that sublime and inge-

" nious, that elegant and beautiful, but at the

" same time fanciful writer, were by no means

" suited to the sober spirit of enquiry cultivated

" by Sir John Pringle. Indeed, whatever at-

" tention he might have paid, in his earlier

" days, and when he was Professor of Ethics at

" Edinburgh, to metaphysical disquisitions, he

" lost all regard for them in the latter part of

" his life."

Dr. Kippis does not conceal from his readers, that Sir John Pringle had not much fondness for poetry. He had not even any distinguished relish for the immortal Shakespeare. As an excuse for this want of poetical taste, the Doctor, who professes himself one of the warmest admirers of our great dramatist, observes that the mind of Sir John Pringle was too closely occupied by philosophical enquiries to have much leisure or inclination for attending to the operations of the imagination.

Though Sir John was not fond of poetry, his biographer relates of him, that he had a great affection for music, of which he was not merely an admirer, but became so far a practitioner titioner in it, as to be a performer on the violoncello, at a weekly concert, given by a ſociety of gentlemen at Edinburgh. He is ſaid, however, to have neglected muſic, as he advanced in years.

In his youth he had not been neglectful of philological enquiries. We are told he paid a great attention to the French language, and was fond of Voltaire's critical writings. Dr. Kippis adds, that among all his other purſuits, Sir John Pringle never forgot the ſtudy of the Engliſh language. This he regarded as a matter of ſo much conſequence, that he took uncommon pains with reſpect to the ſtyle of his compoſitions.—During the latter part of his life, he devoted much of his time to the ſtudy of divinity.

Paſſing from the intellectual to the moral character of Sir John Pringle, his biographer obſerves, that the ruling feature of it was integrity. " All his acquaintance," obſerves Dr. Kippis, " with one voice agree that there never " was an honeſter man. He was equally diſtin" guiſhed by his ſobriety. He told Mr. James " Boſwell, that he had never in his life-time " been intoxicated with liquor."

He was ardent and ſteady in his friendſhips. He paid a very reſpectful attention to thoſe who were honoured with his eſteem, and to ſuch ſtrangers as came to him well recommended. Dr. Kippis acknowledges, however, that he had, at times, ſomewhat of a dryneſs and reſerve in his behaviour, when he was not perfectly pleaſed with the perſons who were introduced to him, or who happened to be in his company.

In the latter part of his life he ſometimes nodded in company. This, we are told, was owing to his having for many years been ſo remarkably troubled for want of reſt, that there was ſcarcely a ſingle night, in which he did not lie awake for ſeveral hours. To this wakefulneſs Dr. Kippis imputes a certain weariſomeneſs and reſtleſſneſs that hung about him, and which he ſought to remove by changes of ſituation. To the ſame cauſe likewiſe, he thinks may be aſcribed, if, in any reſpect, Sir John did not ſuſtain the infirmities of age with that full fortitude and dignity of mind, which, tho' always deſireable, cannot even by the beſt characters always be attained.

Dr. Kippis is at conſiderable pains to inveſtigate the religious character of his author. " He " was another inſtance," we are told, " of " thoſe

" those illustrious philosophers, who have not

" been ashamed of religion; and added another

" name to the catalogue of the excellent and

" judicious persons, who have gloried in being

" rational Christians."

" Such having been the character and emi-

" nence of Sir John Pringle," observes our author, " it was highly proper that his name should

" be recorded among the worthies of Westmin-

" ster Abbey. Accordingly, under the direc-

" tion, and at the expence, of his nephew and

" heir, a monument is preparing, of which Mr.

" Nollekens is sculptor, and for which an Eng-

" lish inscription is intended."—We may add, that since the publication of the work, the monument in question has been erected.

We shall conclude this article with the following elegant inscription, which the Doctor informs us was written by a friend of Sir John Pringle. We shall venture to add, that this classical friend is now pretty generally, and we believe with good reason, supposed to be Sir George Baker.

M. S.

Viri egregii JOHANNIS PRINGLE Baronetti;

Quem exercitus Britannicus,

Celsissima Walliæ Principessa,

Regina sereniſſima,

Ipſius denique Regis Majeſtas,

Medicum ſibi comprobavit

Experientiſſimum, ſagacem, ſtrenuum:

Quem, ſtudiis academicis florentem,

Edinburgenſes olim ſui

In cathedrâ diſciplinæ ethicæ dicatâ

Adhuc juvenem collocarunt;

Quem poſteà, ætate ac ſcientiâ provectum,

Primùm perhonorifico ornavit prœmio,

Deìnde ad ſummam apud ſe dignitatem evexit

Societas Regia Londinenſis.

Qualis fuerit medendi artifex,

Quali rerum comprehenſione præditus,

Materiem ſuam multiplicem

Quam ſcientèr explicuerit et illuſtraverit,

Scripta viri doctiſſimi teſtentur

Per Europam omnem diſſeminata,

Nec foris minùs quam domi nota.

Quâ autem fide et integritate fuerit,

Quam veri tenax et inimicus fraudi,

Quam conſtans Supremi Numinis cultor,

Ii, quibuſcum vixit,

Teſtes ſunto.

Exceſſit e vitâ, &c.

VI. *The*

